# Revisiting G3BP1 as a RasGAP Binding Protein: Sensitization of Tumor Cells to Chemotherapy by the RasGAP 317–326 Sequence Does Not Involve G3BP1

**DOI:** 10.1371/journal.pone.0029024

**Published:** 2011-12-19

**Authors:** Alessandro Annibaldi, Aline Dousse, Sophie Martin, Jamal Tazi, Christian Widmann

**Affiliations:** 1 Department of Physiology, University of Lausanne, Lausanne, Switzerland; 2 Institut de Génétique Moleculaire de Montpellier UMR 5535, IFR 122, Centre National de Recherche Scientifique, Montpellier, France; Hungarian Academy of Sciences, Hungary

## Abstract

RasGAP is a multifunctional protein that controls Ras activity and that is found in chromosomal passenger complexes. It also negatively or positively regulates apoptosis depending on the extent of its cleavage by caspase-3. RasGAP has been reported to bind to G3BP1 (RasGAP SH3-domain-binding protein 1), a protein regulating mRNA stability and stress granule formation. The region of RasGAP (amino acids 317–326) thought to bind to G3BP1 corresponds exactly to the sequence within fragment N2, a caspase-3-generated fragment of RasGAP, that mediates sensitization of tumor cells to genotoxins. While assessing the contribution of G3BP1 in the anti-cancer function of a cell-permeable peptide containing the 317–326 sequence of RasGAP (TAT-RasGAP_317–326_), we found that, in conditions where G3BP1 and RasGAP bind to known partners, no interaction between G3BP1 and RasGAP could be detected. TAT-RasGAP_317–326_ did not modulate binding of G3BP1 to USP10, stress granule formation or *c-myc* mRNA levels. Finally, TAT-RasGAP_317–326_ was able to sensitize G3BP1 knock-out cells to cisplatin-induced apoptosis. Collectively these results indicate that G3BP1 and its putative RasGAP binding region have no functional influence on each other. Importantly, our data provide arguments against G3BP1 being a genuine RasGAP-binding partner. Hence, G3BP1-mediated signaling may not involve RasGAP.

## Introduction

There is an ongoing need to improve current anti-tumor regimens to reduce the rate of death due to cancer. In this context, we discovered earlier that the caspase-3-generated RasGAP N-terminal fragment (RasGAP_158–455_), called N2, was able to selectively sensitize cancer cells, but not healthy cells, to genotoxin-induced apoptosis [1]. RasGAP amino acids 317 to 326 within fragment N2 were found to carry this sensitizing activity [2]. A cell-permeable peptide containing this sequence (the so-called TAT-RasGAP_317–326_ peptide) was then generated [2]. This peptide potently enhances the efficacy of genotoxins to selectively kill cancer cells, both in *in vivo* [3] and *in vitro* [2] settings. TAT-RasGAP_317–326_ does not induce apoptosis by itself making it a pure sensitizer compound [2]–[4]. The understanding of its mode of action is of particular relevance in the context of the mechanisms allowing cancer cells to resist apoptosis. It is known that TAT-RasGAP_317–326_ favors genotoxin-induced mitochondrial outer membrane depolarization (MOMP) and caspase-3 activation [5]. The RasGAP-derived peptide requires a functional p53/PUMA axis to induce its genotoxin-sensitization effect [5]. However, this might only reflect the fact that genotoxins require the p53/PUMA axis to optimally kill cancer cells [6], [7]. At present, the direct molecular target(s) of TAT-RasGAP_317–326_ are unknown and the cellular events underlying its sensitizing properties are only minimally understood.

GAP SH3 Binding Protein 1 (G3BP1) is one of the molecules described to interact with RasGAP. This was first reported by Parker *et al.* in 1996 [8] who identified and cloned a molecule able to bind to the SH3 domain of RasGAP. Incidentally, this interaction only took place in serum-stimulated cells. The binding between RasGAP and G3BP1 could be prevented by a peptide corresponding to sequence 317–326 found within the RasGAP SH3 domain. These data were corroborated by two other reports showing that G3BP1 binds to RasGAP in proliferating cells [9] and that the G3BP1 domain responsible for these binding was the nuclear transfer factor 2 (NTF2)-like domain, located at its N-terminus [10]. This domain was also described to mediate the binding of the yeast orthologue of G3BP1 (Bre5) to the Ubp3 deubiquitinating enzyme [11]. G3BP1 seems not to be a substrate of USP10, the Ubp3 mammalian orthologue, but it appears to inhibit the capacity of USP10 to cleave ubiquitin chains [12]. The C-terminal portion of G3BP1 contains two canonical RNA recognition motifs (RRMs) indicating that G3BP1 has RNA-binding capacities. Indeed G3BP1 was reported to co-immunoprecipitate with mRNAs and to bind to and cleave the 3′ untranslated region (3′UTR) of the *c-myc* mRNA *in vitro* [9]. Interestingly, the endoribonuclease activity of G3BP1 is governed by its phosphorylation status. In proliferating cells, when G3BP1 is hypo-phosphorylated, it loses its ability to cleave mRNA whereas in quiescent cells, when it is hyper-phosphorylated, it does cleave mRNAs [9]. This observation suggested a possible role of G3BP1 in coupling extra-cellular stimuli to mRNA stability. This hypothesis is supported by the finding that G3BP1 is implicated in stress granule (SG) assembly [13]. SGs correspond to cytoplasmic loci where mRNAs are stored during stress conditions and where the decision to degrade or convert them into translationally active mRNA protein complexes (mRNPs) is taken once the stress has subsided [14]–[16]. Formation of stress granules in cells may inhibit apoptosis [17].

The observation that G3BP1 binds to RasGAP on the very same sequence that mediates the tumor sensitizing activity of fragment N2 and the fact that G3BP1 is over-expressed in some cancer cells made G3BP1 a good candidate for the TAT-RasGAP_317–326_ peptide ability to lower the resistance specifically in cancer cells. We therefore investigated whether the known functions attributed to G3BP1 could be modulated by TAT-RasGAP_317–326_ and whether G3BP1 was required for the peptide to sensitize tumor cells to genotoxin-induced apoptosis.

## Materials and Methods

### Cell lines and transfection

U2OS (LGC Promochem; ATCC n° HTB-96), HCT116 [18], HEK293T [19], HeLa cells (LGC Promochem; ATCC n° CCL-2), and CCL39 cells (LGC Promochem; ATCC n° CCL-39), as well as wild-type and G3BP1 knock-out mouse embryonic fibroblasts (MEFs) [20] were maintained in DMEM (Invitrogen reference n°61965) containing 10% fetal calf serum (GIBCO/BRL reference n°10270-106, lot n°41Q6001K) at 37°C and 5% CO_2_. HEK293T and U2OS cells were transfected using the calcium/phosphate precipitation procedure [2], [21].

### Buffers

The composition of phosphate buffered saline (PBS) is 116 mM NaCl, 10.4 mM Na_2_HPO_4_, 3.2 mM KH_2_PO_4_ (pH 7.4). The Stag lysis buffer is made of 50 mM Hepes pH 7.4, 150 mM NaCl, 1.5 mM MgCl_2_, 1 mM EGTA pH 8.0, 10% glycerol, 1% Triton X-100, and is supplemented with one tablet of EDTA-free inhibitor (Roche) per 50 ml. The composition of sample buffer 2X is 25 mM 2-amino-2-(hydroxymethyl)-1,3-propanediol (Tris) HCl pH 6.5, 10% glycerol, 6% SDS, 0.02% of bromophenol blue and 100 mM freshly added dithiothreitol (DTT). The 1% Triton X-100 lysis buffer is made of 20 mM Hepes pH 7.4, 150 mM NaCl, 1% Triton X-100, 1 mM MgCl_2_, 1 mM EGTA and 1 mM NaVO_4_ supplemented with one tablet of EDTA-free inhibitor [Roche] per 50 ml. The RIPA-like lysis buffer is made of 50 mM Tris-HCl pH 7.4, 150 mM NaCl, 1% NP-40, 0.25% deoxycholic acid, 1 mM EGTA and 1 mM NaVO_4_ supplemented with one tablet of EDTA-free inhibitor (Roche) per 50 ml. The composition of sample buffer 5X is 250 mM Tris-HCl pH 7, 10% SDS, 30% glycerol, 5% β-mercaptoethanol. Tris buffer saline (TBS) is made of 20 mM Tris base, 130 mM NaCl, pH 7.6.

### Antibodies

The monoclonal antibody specific for the hemagglutinin (HA) tag was purchased as ascites from BabCo (reference n°MMS-101R). This antibody was adsorbed on HeLa cell lysates to decrease non-specific binding as described previously [22]. Mouse anti-human G3BP1 was from BD Transduction Laboratories (reference n°611127). The rabbit polyclonal anti human TIA-1 antibody was from Santa Cruz (reference n°sc-28237) The anti S-Protein HRP-conjugated antibody was from Novagen (reference n°69047). The anti-GST antibody was from Upstate (reference n°06-332). The polyclonal rabbit anti-c-Myc antibody was from Cell Signaling (reference n°9402). The polyclonal rabbit anti-RasGAP antibody directed at the Src homology (SH) domains of RasGAP was from Enzo Life Sciences (reference n°ALX-210-860-R100). The monoclonal mouse anti-SV40 large T antigen was from BD Pharmingen (reference n°554149). The rabbit anti-USP10 antibody was provided by Dr. Olivier Staub (University of Lausanne, Switzerland). The monoclonal mouse anti-V5 antibody was from Invitrogen (reference n°46-0705). Secondary antibodies were donkey anti-mouse fluorescein-conjugated antibody and donkey anti-rabbit Cy3-conjugated antibody (Jackson ImmunoResearch, reference n°715-095-1507 and 711-165-152, respectively).

### Plasmids

The extension .*dn3* indicates that the backbone plasmid is pcDNA3 (#1) (Invitrogen). *S·tag* corresponds to the first 15 amino acids of the S-peptide of RNase A (amino acids 1–15 of RNAse A). The S·tag is able to bind with high affinity to the S protein (amino acid 21–124 of RNAse A) [23], [24]. **TRIP-PGK-IRESNEO-WHV** (#350) is a lentiviral vector bearing the neomycine resistance. The **pEGFP-C1** plasmid (#6) encodes the green fluorescent protein and is from Clonetech. The **hG3BP1.dn3** plasmid (#322) encodes human G3BP1 [8]. **HA-rRhoGAP.prc** (#196) encodes the HA-tagged form of rat p190RhoGAP. The **6xHis-Stag-mUSP10.dn3** plasmid (#646) encoding the poly-histidine- and S-tagged form of mouse USP10 was described earlier [25]. **HA-hRasGAP.dn3** (#118), previously called HA-GAP.dn3 [22], encodes the HA-tagged form of human RasGAP. **HA-hRasGAP[158–455].dn3** (#145), previously called HA-N2.dn3 [22], encodes the HA-tagged form of fragment N2. **HA-hRasGAP[1–455](D157A).dn3** (#352), previously called N-D157A.dn3 [22], encodes the HA-tagged caspase-3-resistant form of fragment N. **V5-hRasGAP[3–455](D157A).dn3** (#585) encodes a V5-tagged version of the caspase-3-resistant form of fragment N that lacks its first two methionine residues (to prevent potential internal translation events). It was constructed by PCR amplifying HA-hRasGAP[1–455](D157A).dn3 with oligonucleotide #559 [AA (feeder sequences) GGTACC (KpnI site) GCCACC (Kozak sequence) ATG GGA AAA CCA ATA CCA AAT CCA CTA CTA GGC CTA GAC AGT ACA (V5 tag) GCG GCC GAG GCC GGC AGTG (sequences complementary to RasGAP from third amino acid on; nucleotides 124–133 of the human RasGAP mRNA; entry M23379)] and oligonucleotide #560 [GCA ACG AAG TGG GCA GTT TG (sequences lying within the human RasGAP mRNA downstream of the SacII site; nucleotides 489–469 of human RasGAP mRNA; entry M23379)]. The resulting 427 bp PCR fragment was cut with KpnI and SacII and subcloned in HA-hRasGAP[1–455](D157A).dn3 opened with the same enzymes. **V5-hRasGAP[3-1931].dn3** (#686) encodes a V5-tagged version of human RasGAP that lacks its first two methionine residues (to prevent potential internal translation events). It was made by subcloning the SpeI/SacII fragment of V5-hRasGAP[3–455](D157A).dn3 into HA-hRasGAP.dn3 opened with the same enzymes. **HA-hG3BP1.dn3** (#541) encodes an HA-tagged version of human G3BP1. It was produced by PCR amplifying the hG3BP1.dn3 plasmid with oligonucleotide #501 [AAAA (feeder sequences), GGATCC (BamHI site), GGATCC (Kozak sequence), ATG GGC TAC CCG TAC GAC GTG CCG GAC TAC GCT TCT (HA tag), ATG GTG ATG GAG AAG CCT AG (nucleotides 172–191 of the human G3BP1 mRNA; NCBI entry NM_005754) and oligonucleotide #502 [TTTTT (feeder sequences), GTCGAC (SalI site) TTC ACT GCC GTG GCG CAA GCC CCC TTC (nucleotides 1573–1547 of the human G3BP1 mRNA; NCBI entry NM_005754). The resulting 1465 bp fragment was blunted with T4 DNA polymerase (Promega reference n°M421A), digested with BamHI and subcloned into pcDNA3 opened with BamHI and EcoRV. **Stag-GFP.dn3** (#647) encodes an S-tagged form of the green fluorescent protein (GFP). It was generated by PCR amplifying plasmid pEGFP-C1 with oligonucleotide #686 [AAAAAA (feeder), GGATCC (BamHI site), GCCACC (Kozak sequence), ATG AAA GAA ACC GCT GCT GCT AAA TTC GAA CGC CAG CAC ATG GAC AGC (S·tag), ATG GTG AGC AAG GGC GAG GA (first 20 coding nucleotides of GFP)] and with oligonucleotide #687 [AAA (feeder), CCG TCG ACT GCA GAA TTC GAA GC (nucleotides of pEGFP-C1 surrounding the EcoRI site; underlined)]. The amplified PCR fragment was then digested with BamHI and EcoRI and subcloned into pcDNA3 opened with the same enzymes. **Stag-hRasGAP[158–455].dn3** (#754) encodes the S-tagged form of fragment N2 and was constructed by PCR amplifying plasmid HA-hRasGAP[158–455].dn3 with oligonucleotide #796 [TAAGCAG (feeder sequence), AAGCTT (HindIII), CTCGAG (XhoI), CCACC (Kozak sequence; the last nucleotide of the XhoI recognition site provides the G at the −6 Kozak position), ATG GCG (start codon and alanine codon), AAA GAA ACC GCT GCT GCT AAA TTC GAA CGC CAG CAC ATG GAC AGC (S·tag) TCT CTG GAT GGA CCA GAA TA (first 21 base pairs of fragment N2) and oligonucleotide #679 [GCA TTT AGG TGA CAC TAT AG (nucleotides 1018–999 of pcDNA3)]. The resulting 1027-base pairs PCR fragment was then digested with HindIII and subcloned in HA-hRasGAP[158–455].dn3 opened with the same enzyme. **SV40LargeTantigen.pBABE-puro** (#731) encodes the SV40 large T antigen (Addgene; plasmid 13970). **SV40LargeTantigen.lti-neo** (#738) similarly encodes the large T antigen but in a lentiviral expression vector. It was constructed by subcloning the BamHI 2187 base pairs fragment of SV40LargeTantigen.pBABE-puro into TRIP-PGK-IRESNEO-WHV opened with the same enzyme. **Non-target.pLKO-puro** (#584) corresponds to a pLKO.1-puro lentiviral expression vector containing a shRNA insert that does not target human and mouse genes (Sigma Aldrich, reference n°SHC002). **shRNA-hG3BP1.pLKO-puro** (#641) encodes a shRNA targeting a sequence within the 3′UTR of the human G3BP1 mRNA (nucleotides 1976–1997 of entry NM_005754). It was purchased from Sigma Aldrich. **6xHis-hRasGAP[279–343].pET28** (#544) encodes a histidine-tagged form of the SH3 domain of RasGAP. It was constructed as follows. HA-hRasGAP(no 3′UTR).dn3 (#424) [1] was amplified by PCR using oligonucleotide #505 [AT (feeder) CATATG (NdeI site) AGA AGG CGT GTA CGA GCT AT (human RasGAP; nucleotides 959-978 of NCBI entry M23379)] and oligonucleotide #506 [AT (feeder) GCGGCCGC (NotI site) CTA (stop codon) CCG GCC CAC CTC CTC TAC TA (human RasGAP; nucleotides 1143–1122 of NCBI entry M23379)]. The amplified fragment was cut with the NdeI and NotI restriction enzymes and the resulting 192 base pair fragment was subcloned into vector pET-28a(+) (#543) opened with the same enzymes. A PCR-generated A to G silent mutation was found at position 1129 (numbering based on NCBI entry M23379) in 6xHis-hRasGAP[279–343].dn3. **His-TAT-GFP** (#130) encodes a fusion protein made of a stretch of 6 histidine residues, the TAT sequence, and GFP.

### S•TAG pull down

Two millions U2OS cells were seeded in a six-well plate and the next day transfected using the calcium/phosphate precipitation procedure. After an additional 24-hour period, cells were lysed in Stag lysis buffer and 1 mg of the lysates were incubated with 1 µl of biotinylated S-protein (Novagen; reference n°69218) for 3 hours at 4°C. Thirty µl of streptavidin beads (GE Healthcare Bio-Sciences; reference n°17-5113-01) were then added to the samples and the incubation resumed for an additional 1-hour period. Pull down complexes were then washed 3 times with PBS, 1% NP40 and solubilized in 30 µl of sample buffer 2X.

### Immunoprecipitation

One million cells (either CCL39 or U2OS) were seeded in 10-cm plates and 24 hours later the cells were lysed in 1% Triton X-100 lysis buffer. Alternatively, one million HEK 293T cells were seeded in 10-cm plates and 24 hours later they were transfected using the calcium/phosphate precipitation procedure. After an additional 24-hour period they were lysed in RIPA-like lysis buffer. Protein content was measured by Bradford. Seven hundreds µg of total protein were immunoprecipitated with 1 µg of the anti-RasGAP antibody overnight at 4°C with rotation at 9 rpm. Twenty µl of G protein sepharose beads (GE Healthcare; reference n°17-0618-01) were then added for an additional 2 hour period. Immunocomplexes were then washed three times with washing buffer (50 mM Tris-HCl pH 7.4, 150 mM NaCl, 1% NP-40) and solubilized in 30 µl of sample buffer 2X. Samples were heated 10 minutes at 95°C before loading.

### Quantitative PCR

RNA was isolated with the “High Pure RNA isolation kit” (Roche; reference n° 11828665001) according to the manufacturer's instruction. The RNA was then reverse-transcribed with the “Transcriptor high fidelity cDNA kit” (Roche; reference n°05 091 284 001) as per the manufacturer's instruction. Quantitative PCR assays were carried out on a real-time PCR detection system (iQ5; Bio-Rad) using iQ SYBR Green Supermix (Bio-Rad; reference n° 170–8862) using primers at a 500 nM concentration. The sequences of the *c-myc* specific primers were GGA CGA CGA GAC CTT CAT CAA (oligonucleotide #728, nucleotide 926–946 of *c-myc* mRNA, NCBI entry: NM_002467.3) and CCA GCT TCT CTG AGA CGA GCT T (oligonucleotide #729, nucleotide 996–1017 of *c-myc* mRNA, NCBI entry: NM_002467.3). The 18S ribosomal RNA was used for normalization. The primers used to amplify this RNA were GCA ATT ATT CCC CAT GAA CG (oligonucleotide #774, nucleotide 1617–1636, NCBI entry: NR_003278.1) and GGC CTC ACT AAA CCA TCC AA (oligonucleotide #775, nucleotide 1720–1739, NCBI entry: NR_003278.1).

### Western blotting

Two hundred thousand cells were seeded in six-well plates and 24 hours later they were subjected to the treatments indicated in the figures after which they were lysed in sample buffer 5X. Proteins were quantitated using the Bradford method. Equal amounts of proteins were subjected to SDS-PAGE and then transferred onto a nitrocellulose membrane (Biorad; reference n°162 0115). The membranes were blocked with TBS containing 0.1% Tween-20 and 5% non-fat milk and incubated overnight at 4°C with the indicated primary antibodies used at 1∶1000 dilution. Blots were then washed with TBS-Tween 0.1%, incubated with the appropriate secondary antibody (1∶5000 dilution) 1 hour at room temperature and subsequently visualized with the Odyssey infrared imaging system (LICOR Biosciences, Bad Homburg, Germany).

### Lentivirus

Recombinant lentiviruses were produced as described [26]. Briefly, HEK293T cells were co-transfected using the calcium phosphate DNA precipitation method [21] with 50 µg of the lentiviral vector (TRIP-PGKATGm-MCS-WHV) containing the cDNA of interest (i.e. G3BP1 shRNA), 2.5 µg of the envelope protein–coding plasmid (pMD.G), and 7.5 µg of the packaging construct (pCMVDR8.91). Two days after transfection, the virus-containing medium was harvested. Infection of the cells was performed as follows. Hexadimethrine bromide (Polybrene; Sigma; reference n°52495) was added to cells cultured in six-well plates at a final concentration of 5 µg/ml followed by the addition of the lentivirus. The plates were then centrifuged 45 minutes at 800 *g* and placed 24 hours at 37°C in a 5% CO_2_ humidified atmosphere. The medium was then replaced with fresh medium, and the cells were further cultured for an additional 48 hour period before being used in specific experiments.

### Immunocytochemistry

Two hundred thousand cells were seeded in six-well plates containing glass coverslips and 24 hours later they were transfected with the calcium/phosphate precipitation procedure. One day post-transfection, the cells were fixed as follows (all steps were performed at room temperature). The cells on coverslips were washed with 4 ml of PBS, fixed with 3 ml PBS, 3% formaldehyde, 3% sucrose for 10 minutes, washed thrice with PBS, permeabilized with 2 ml of PBS, 0.2% Triton X-100 for 10 min, washed thrice with PBS, and incubated 30 minutes with 3 ml of filtered serum-containing culture medium. After three additional PBS washes, the coverslips were incubated for 1 hour with the primary antibody diluted in DMEM, 10% newborn calf serum. The coverslips were washed 3 times over 30 minutes in PBS and then incubated 1 hour with a 1/100 dilution of labeled secondary antibodies in DMEM, 10% newborn calf serum. The coverslips were washed 3 more times in PBS and labeled when indicated with 10 µg/ml Hoechst 33342 (Molecular Probe) before being mounted (Vectashield mounting medium, Vector laboratories Inc). Confocal images were captured with a Leica SP5 AOBS confocal microscope.

### Determination of G3BP1 nuclear content

Confocal images were converted to tif format and opened with the Adobe Photoshop Elements 5.0 software. Pictures of Hoechst-stained cells were used to create masks of the nuclei that were then overlaid on the pictures of G3BP1-stained cells in order to delimitate the nuclear area. The nuclear G3BP1 signal image was then opened using the ImageJ software (1.34n version) and the nuclear signal was quantitated. Similarly, the total G3BP1 staining was quantitated for each picture with the ImageJ software (in this case the cell contours were manually drawn). The nuclear G3BP1 signal was calculated as the percentage of the total G3BP1 cell staining.

### Peptides

TAT and TAT-RasGAP_317–326_ are retro-inverso peptides (i.e. synthesized with D-amino acids in the opposite direction compared to the natural sequence). The TAT moiety corresponds to amino acids 48–57 of the HIV TAT protein (RRRQRRKKRG) and the RasGAP_317–326_ moiety corresponds to amino acids 317–326 of the human RasGAP protein (DTRLNTVWMW). These two moieties are separated by two glycine linker residues in the TAT-RasGAP_317–326_ peptide. The peptides were synthesized at the Department of Biochemistry, University of Lausanne, Switzerland, using FMOC technology, purified by HPLC and tested by mass spectrometry.

### Protein production and purification

Plasmids coding for recombinant proteins were expressed in BL21 *E. coli*. Bacteria were grown overnight in lysogeny broth (LB) medium (casein enzymic hydrolysate 10 g/l, yeast extract 5 g/l, NaCl 170 mM, pH 7.5). The induction of the recombinant proteins was performed by the addition of 0.1 mM isopropyl-beta-thio-galactoside (IPTG) to the culture medium when it reached on optical density at 600 nm of 0.6. After 4 hours of induction, the cells were harvested and resuspended in 5 ml Buffer A [HEPES 50 mM, magnesium acetate 200 mM, NaCl 500 mM, Triton X-100 0.1%, lysozyme 2 mg/ml, 0.5% β-mercaptoethanol and 10 µg/ml DNaseI, supplemented with one tablet of EDTA-free inhibitor (Roche) per 50 ml]. In order to fully lyse the cells, the suspension was sonicated (Heischer DmBH sonicator, 0.9 cycles per second, 80% amplitude) 4 times 30 seconds on ice and then centrifuged 20 minutes at 9'000 g at 4°C. A sample of the lysate was kept to verify the induction of the protein. GST-tagged proteins were then purified. The glutathione-sepharose beads (Amersham Pharmacia Biotech; reference n°17-0756-01) were washed 3 times with PBS and then incubated with the lysate at 4°C with rotation (Labinco rotary mixer, 12 rpm). The beads were washed thrice with PBS. To elute the recombinant protein, the beads were incubated with 500 µl of glutathione elution buffer (50 mM Tris pH 8, 10 mM reduced glutathione [Sigma reference n°G-4251]) for 15 minutes at 4°C with rotation. The beads were then pulled down at 4'000 g and the supernatant was collected. Histidine-N2 recombinant proteins were produced as described above, except that nickel beads were used (Ni-NTA agarose, Qiagen; reference n°1000632) instead of glutathione-Sepharose beads. The elution step was then performed with buffer A (see above) supplemented with 25 mM imidazole (Sigma reference n°I2399).

### Statistics

All the statistical analyses were done with Microsoft Excel (XP edition) using the unpaired Student's t test. Significance is indicated by an asterisk when P<0.05/n, where P is the probability derived from the t test analysis and n is the number of comparisons done (Bonferroni correction).

### *In silico* protein docking assays

The G3BP1 NTF2 domain structure (PDB entry #2Q90) was first refined using Maestro v90211 in order to complete and refine the loops. Molecular dynamics simulations were conducted using the AMBER 11.0 force field from the NAMD 2.7 package. Using AmberTools 11.0, the resulting system was solvated in a rectangular box extending 12 Å around the molecule using TIP3P water molecules. Sodium and chloride ions were added to neutralize the system. Five thousand steps of energy minimization were applied on the entire system. Following minimization, the system was equilibrated with 5'000 steps of water-only molecular dynamics at 150°K, the system was heated from 0 to 300°K for 100 picoseconds. After heating, a 5 nanosecond production simulation was conducted with a 1 picosecond time step at a pressure of 1 bar and a temperature of 300°K. The RasGAP SH3 domain (PDB entry #2JO5) was optimized using the same method. The docking was performed using Hex 5.1 standard parameters. The 150 best poses out of 10'000 were collected and analyzed. The resulting complexes were ranked according to their free interactions energy. The same docking method was applied to the SH3 domain of RasGAP and sequence 190–233 of UBP3, the USP10 yeast orthologue (PDB entry #2QIY).

## Results

### No evidence for a RasGAP-G3BP1 interaction

**Figure 1:**
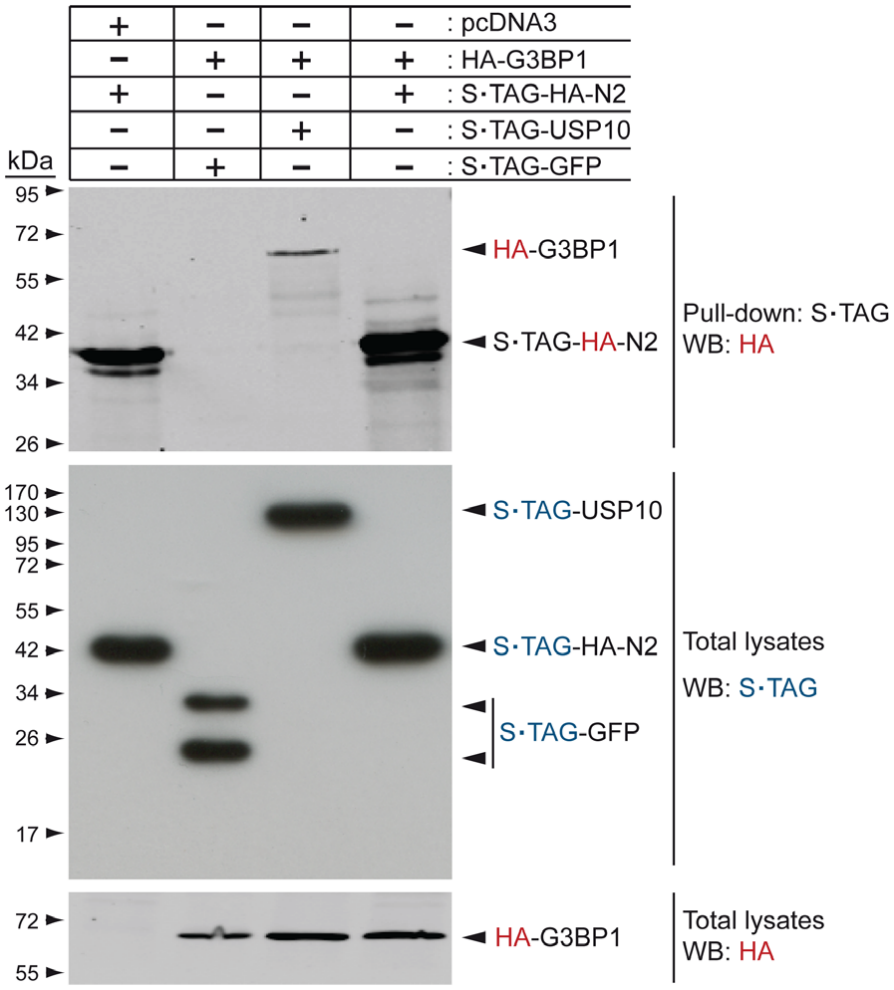
G3BP1 does not bind to RasGAP-derived fragment N2. Lysates (1 mg) from HEK293T cells that had been transfected with the indicated plasmids were subjected to S•TAG pull-down. Pulled-down complexes were then analyzed by Western blotting using an HA-specific antibody. In parallel, 50 µg of total cell lysates were subjected to Western blot analysis using antibodies recognizing HA or the S•TAG.

**Figure 2:**
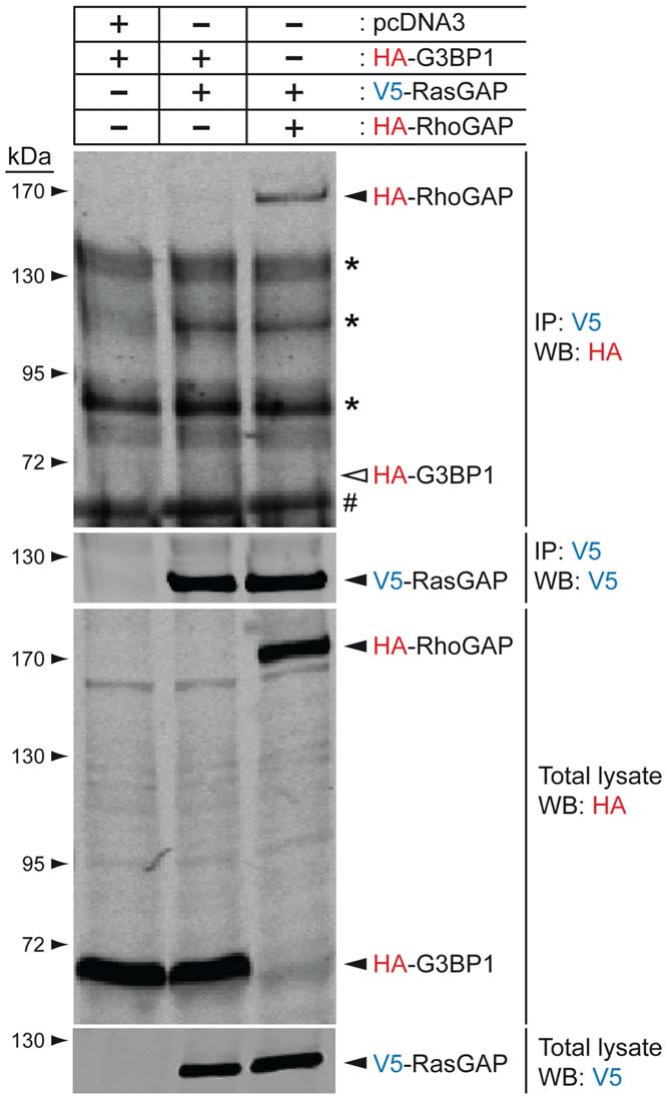
Ectopically-expressed RasGAP and G3BP1 fail to interact in conditions where RasGAP binds to RhoGAP. Lysates (1 mg) from HEK293T cells that had been transfected with the indicated plasmids were immunoprecipitated with an anti-V5 antibody. Immunoprecipitated complexes and cell lysates (50 µg) were analyzed by Western blotting using HA- and V5-specific antibodies. Asterisks: non-specific bands; #: immunoglobulin heavy chains; white arrowhead: expected migration of HA-G3BP1.

**Figure 3:**
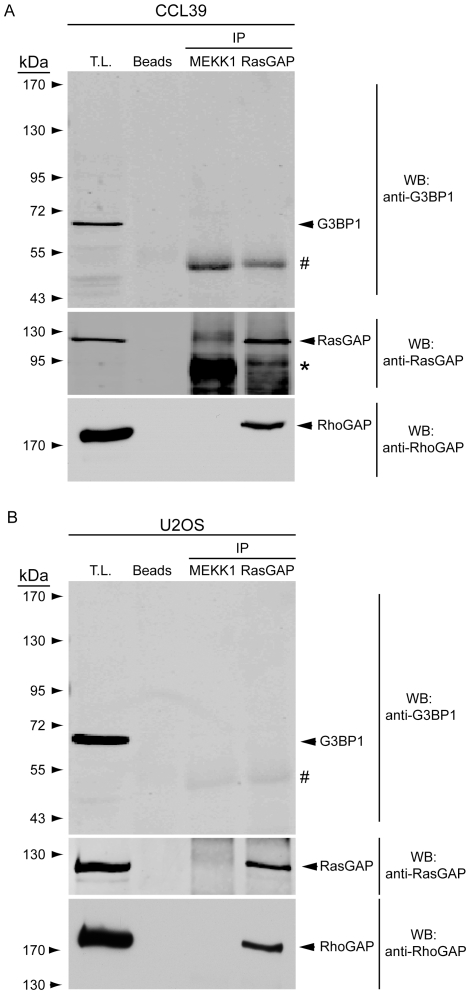
Endogenous RasGAP binds to RhoGAP but does not associate with G3BP1. Non-confluent, exponentially growing CCL39 cells (panel A) or U2OS cells (panel B) were lysed in 1% Triton X-100 lysis buffer and 1 mg of total protein extracts were immunoprecipitated with an anti-RasGAP antibody. Immunoprecipitated complexes and cell lysates (50 µg) were analyzed by Western blotting using G3BP1- and p190 RhoGAP-specific antibodies. T.L.: total lysate; asterisks: non-specific bands; #: immunoglobulin heavy chains.

Previous data indicated that G3BP1 binds to a discrete sequence of RasGAP within its SH3 domain that corresponds to amino acids 317–326 of the protein [8]. As fragment N2 bears this SH3 domain, we postulated that it should allow fragment N2 to bind to G3BP1. However, Figure 1 shows that in HEK 293T cells fragment N2 failed to pull down G3BP1 in conditions where USP10, a deubiquitinating enzyme known to interact with G3BP1 [12], did. It could be argued that the 317–326 sequence is not accessible on fragment N2 while it would be exposed on the parental full-length protein. We therefore assessed if an interaction between RasGAP and G3BP1 could be demonstrated. Figure 2 indicates that RasGAP could not co-immunoprecipitate with G3BP1, while it did with p190 RhoGAP, a known RasGAP binding partner [27], [28]. G3BP1 binding to RasGAP has been reported in the CCL39 (Chinese hamsters lung fibroblast) cell line [9]. Conceivably, this interaction could be cell-type specific. Therefore, we immunoprecipitated RasGAP from both CCL39 cells and U2OS cells. Figure 3 shows that endogenous RasGAP protein interacted with p190 RhoGAP in both cell lines. In these conditions, no interaction could be detected between RasGAP and G3BP1.

### TAT-RasGAP_317–326_ does not affect the binding of G3BP1 to USP10

**Figure 4:**
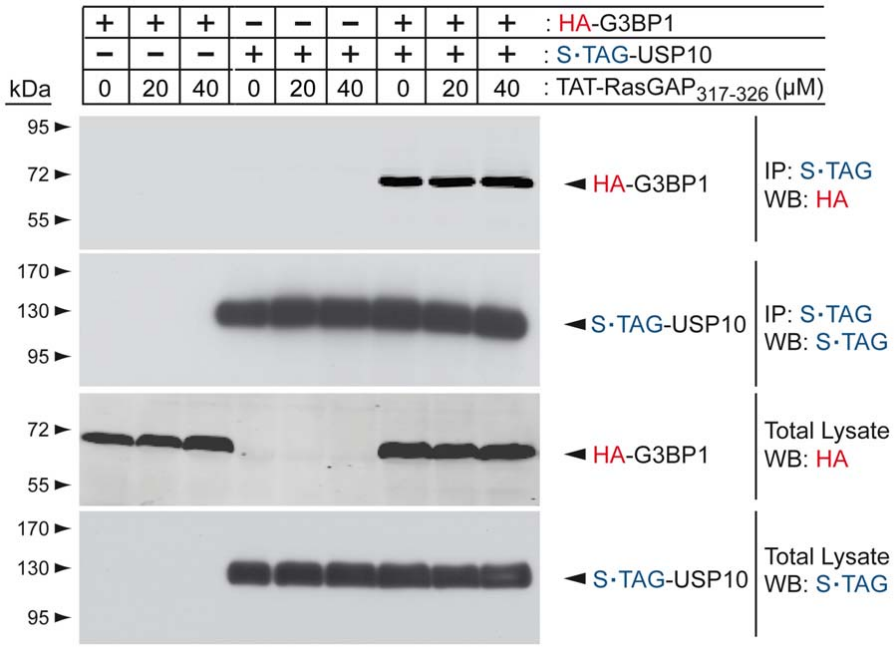
TAT-RasGAP_317–326_ does not affect the binding of G3BP1 to USP10. HEK293T cells were transfected with the indicated plasmids. Eight hours later, they were treated with the indicated concentrations of TAT-RasGAP_317–326_ for an additional 20 hour period at which time they were lysed. Lysates (1 mg) were subjected to S•TAG pull-down. Pulled-down complexes were analyzed by Western blotting using HA- and S•TAG specific antibodies. In parallel 50 µg of total cell lysates were subjected to Western blotting analysis using the same antibodies.

It might be argued that the G3BP1-RasGAP interaction is transient and difficult to detect by the techniques we have used here. This interaction might nevertheless occur and one could hypothesize that TAT-RasGAP_317–326_ sensitizes tumor cells by modulating G3BP1 functions by either inhibiting the binding of RasGAP to G3BP1 or by mimicking the binding of RasGAP to G3BP1. One of the reported functions of G3BP1 is to bind to and inhibit USP10 [12]. We therefore assessed if TAT-RasGAP_317–326_ could prevent the binding of G3BP1 to this protein. Figure 4 shows that the interaction of G3BP1 to USP10 was unaffected by TAT-RasGAP_317–326_, indicating that the peptide does not affect the inhibitory function of G3BP1 on USP10.

### TAT-RasGAP_317–326_ does not impair stress granule formation

**Figure 5:**
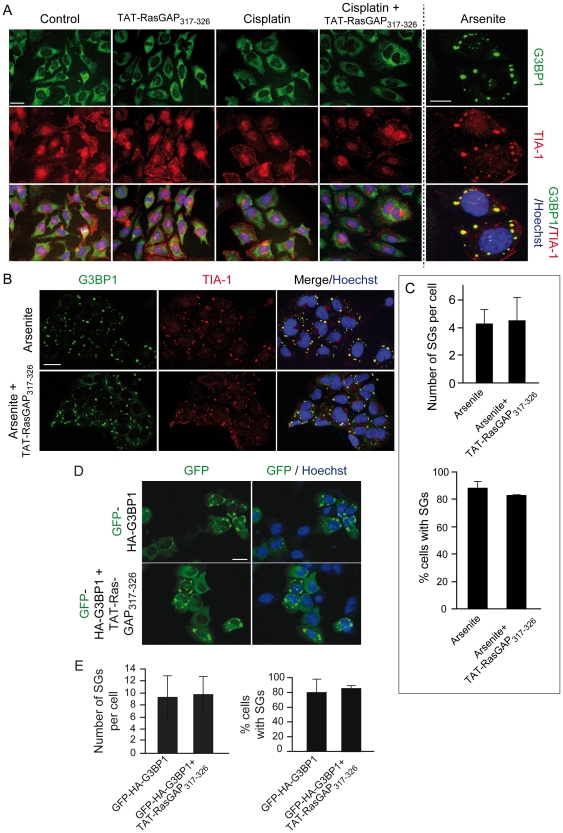
TAT-RasGAP_317–326_ does not affect G3BP1-induced SG formation. **A.** U2OS cells were left untreated or incubated with 15 µM cisplatin, 20 µM TAT-RasGAP_317–326_, or a combination of the two compounds for 22 hours. Alternatively, the cells were treated with 200 µM arsenite for 2 hours. The cells were then processed for immunofluorescence analysis using TIA-1- and G3BP1-specific antibodies. Nuclei were stained with Hoechst 33342. Images were taken with a fluorescent microscope (Nikon Eclipse 90i). Scale bar: 20 µm for the first 4 columns, 5 µm for the last column. **B–C.** U2OS cells were pre-incubated or not with 20 µM TAT-RasGAP_317–326_ for 18 hours and then treated with arsenite (200 µM, 2 hours). The cells were then processed as in panel A. Scale bar: 20 µm. Quantitation of the number of SGs per cells and the percentage of cells with SGs is shown in panel C. **D–E.** U2OS cells were transfected with a plasmid encoding GFP-HA-G3BP1 and 6 hours later were incubated or not with 20 µM TAT-RasGAP_317–326_ for an additional 22 hour period. Cells were then fixed and their nuclei stained with Hoechst 33342. Scale bar: 20 µm. Panel E shows the quantitation of the number of SGs per cells as well as the percentage of cells with SGs.

In response to environmental stress (i.e. heat and oxidative conditions) eukaryotic cells stop the translation of constitutive expressed mRNAs that are then routed to phase-dense cytoplasmic granules called stress granules (SGs). Many RNA-binding proteins participate in SG assembly including G3BP1, which together with TIA-1, is a SG marker [16]. An attractive hypothesis was that TAT-RasGAP_317–326_ mediates its sensitization effect by inhibiting the capacity of G3BP1 to participate in SG formation when cells are subjected to cytotoxic stresses. Therefore we tested if cisplatin was able to induce SG formation in U2OS cells and if TAT-RasGAP_317–326_ could impair this stress-induced response. As shown in Figure 5A, formation of SGs after treatment with arsenite was observed, but cisplatin did not induce SG assembly. Consequently, it is unlikely that the ability of TAT-RasGAP_317–326_ to sensitize U2OS cells to genotoxin-induced apoptosis relies on an effect on SGs. Nevertheless, to assess whether TAT-RasGAP_317–326_, in conditions where SGs are efficiently induced, modulates their assembly, U2OS cells were pre-incubated with TAT-RasGAP_317–326_ and then incubated with arsenite. Figure 5B–C shows that the peptide did not affect the number of SG per cell or the number of cells exhibiting SGs. To directly evaluate whether TAT-RasGAP_317–326_ affects SG formation induced by G3BP1, U2OS cells were transfected with a GFP-tagged form of G3BP1 and incubated or not with the RasGAP-derived peptide. In this case again, the presence of TAT-RasGAP_317–326_ did not alter the formation of SGs (Figure 5D–E). Altogether, these results show, not only that genotoxins do not induce SG formation, but also that TAT-RasGAP_317–326_ does not affect the ability of G3BP1 to mediate the formation of SGs.

### TAT-RasGAP_317–326_ does not sensitize cancer cells through the modulation of *c-myc* mRNA levels

**Figure 6:**
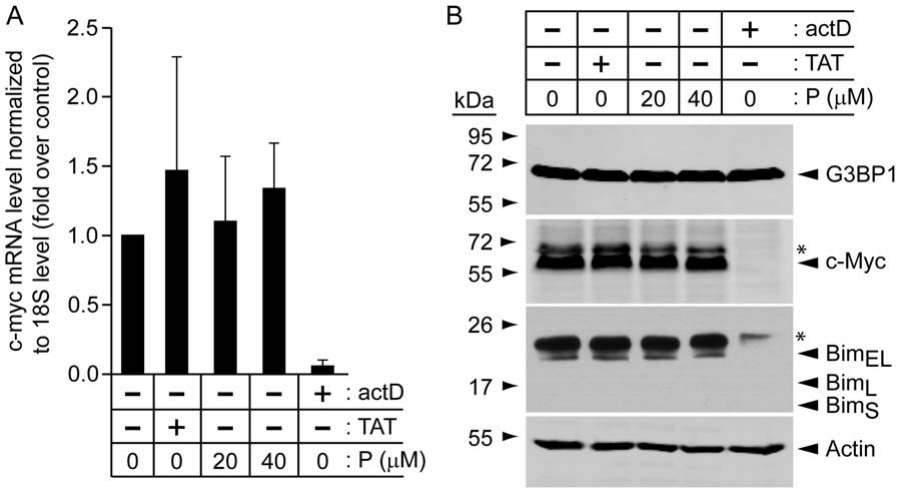
TAT-RasGAP_317–326_ does not affect *c-myc* mRNA and protein levels or expression of the c-Myc target Bim. **A.** HeLa cells were treated or not for 24 hours with 20 µM TAT, 20 or 40 µM TAT-RasGAP_317–326_ (P), or 1 µg/ml actinomycin D (actD). Quantitative RT-PCR was then used to measure *c-myc* mRNA levels. **B.** Alternatively, the cells were lysed and 50 µg of protein extracts were analyzed by Western blotting using the indicated antibodies. Note that the Bim gene encodes three different forms of the protein, the expected migrations of which are indicated. Asterisks indicate non-specific bands.

The endoribonuclease activity of G3BP1 was first reported by its ability to cleave the 3′ untranslated region (UTR) of the *c-myc* transcript [9], [29]. This transcription factor regulates the expression of hundreds of gene controlling many cellular functions including cell survival and cell death [30]. It is an oncogene (one of the first to have been characterized actually) that is deregulated in many cancer types [31]. It plays a role in apoptosis by modulating proteins belonging to the Bcl-2 family, such as the pro-apoptotic BH3 only protein Bim [32], [33]. The 3′ UTR of the *c-myc* mRNA regulates its stability but how it does so is unclear. There are reports indicating that the 3′ UTR favors *c-myc* mRNA decay [34], [35], while another one provides indirect evidence that the 3′ UTR contributes to *c-myc* mRNA stabilization [29]. If TAT-RasGAP_317–326_ modulates the ability of G3BP1 to cleave the *c-myc* mRNA it could affect the sensitivity of cells to apoptosis, in particular if c-Myc protein levels are increased because this can lead to cancer cell apoptosis, potentially via induction of Bim expression [36], [37]. We therefore checked if TAT-RasGAP_317–326_ modulated *c-myc* mRNA levels and whether it affected c-Myc and Bim protein expression. Figure 6 shows that TAT-RasGAP_317–326_ did not modulate *c-myc* mRNA or c-Myc protein levels. Similarly, Bim expression was not affected by the peptide (Figure 6B). The levels of c-Myc and Bim were efficiently decreased by Actinomycin D, a transcription inhibitor, indicating that the experimental conditions used in the figure allow detecting down-modulation of c-Myc and Bim. Finally, G3BP1 protein levels did not appear to be affected by TAT-RasGAP_317–326_ but an effect of the peptide on the transcription or translation of G3BP1 cannot be ruled out as the half-life of G3BP1 is considerably longer than c-Myc or Bim (Figure 6B). Collectively these results do not support the possibility that TAT-RasGAP_317–326_ modulates the endoribonuclease activity of G3BP1 to mediate its tumor sensitization property.

### TAT-RasGAP_317–326_ does not affect G3BP1 subcellular location

**Figure 7:**
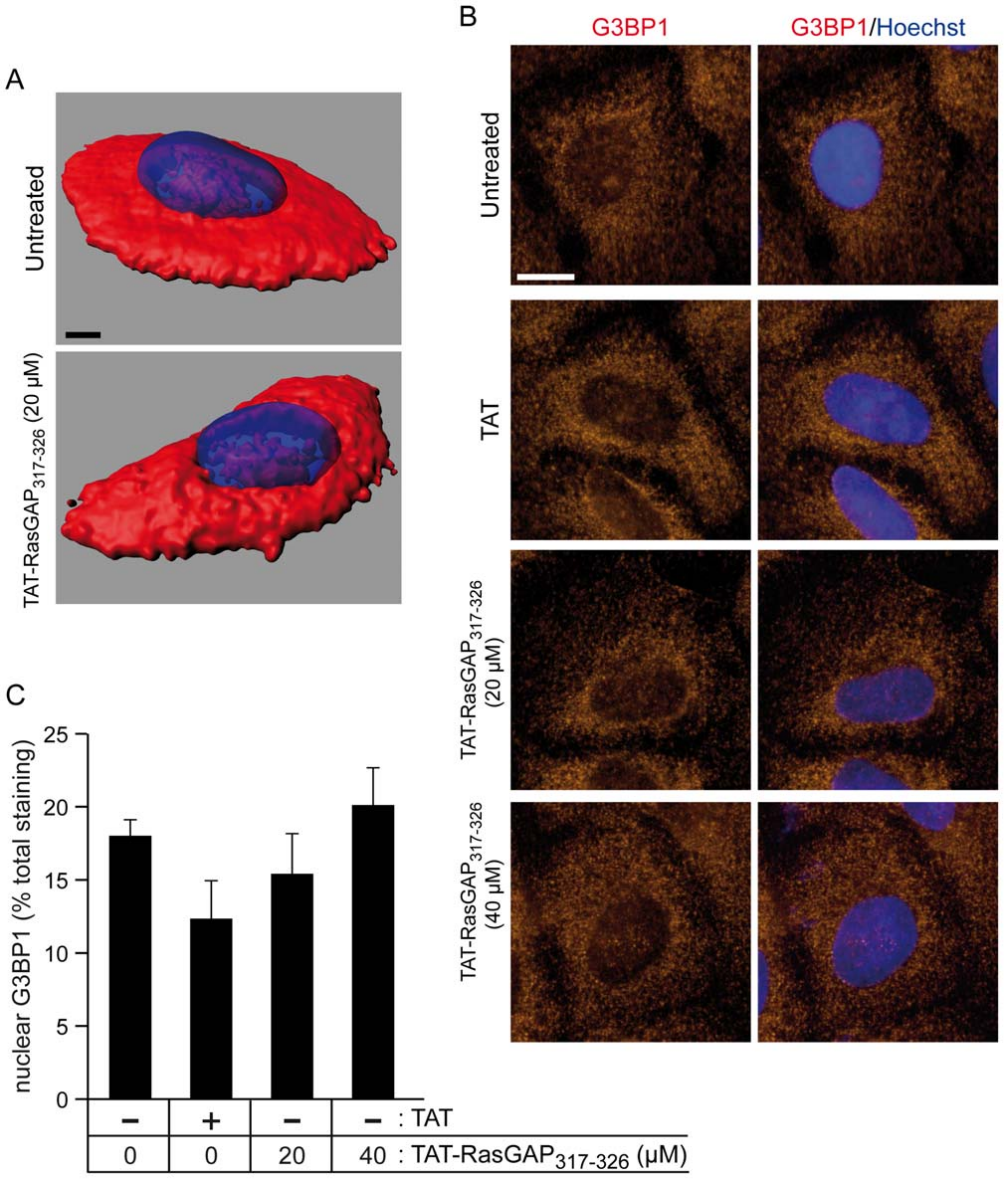
TAT-RasGAP_317–326_ does not affect G3BP1 nuclear localization. A. HeLa cells were left untreated or treated for 18 hours with TAT (20 µM), TAT-RasGAP_317–326_ (20 or 40 µM). Immunocytochemistry against G3BP1 was then performed (the nuclei were stained in blue with the Hoechst 33342 dye) and confocal z-stacks were acquired. Three-dimensional images were then built with the Imaris software. Representative examples of untreated and TAT-RasGAP_317–326_-treated cells are shown (scale bar: 5 µm). Alternatively, images were taken using conventional epifluorescence microscopy (scale bar: 10 µm). Panel B depicts representative examples and panel C shows the corresponding quantifications performed on 80 cells as described in the methods.

Another possibility we wanted to explore concerned G3BP1 localization. It was reported that in quiescent MEFs, G3BP1 relocalizes to the nucleus and that this relocalization modulates its phosphorylation status and endoribonuclease activity [29]. Specifically, when G3BP1 translocates to the nucleus, it becomes phosphorylated on serine 149 and it functions as an active endoribonuclease whereas in proliferating cells, possibly in association with RasGAP, it is dephosphorylated and loses its ability to cleave RNAs [29]. Therefore we assessed whether TAT-RasGAP_317–326_ could alter the sub-cellular location of G3BP1. In HeLa cells, three-dimensional reconstructions of confocal sections indicated that G3BP1 was mainly located on a flat section of the cytoplasm and was absent in areas directly above or below the nucleus (egg-on-a-plate configuration) (Figure 7A). This permitted quantitation of the nuclear G3BP1-specific signal to be performed on conventional epifluorescence images (Figure 7B), which revealed that 15–20% of G3BP1 was localized in the nucleus (Figure 7C). This nuclear location was not affected by the RasGAP-derived peptide however, indicating that the mechanism by which TAT-RasGAP_317–326_ sensitizes tumor cells to genotoxin-induced apoptosis does not rely on modulation of the nuclear G3BP1 content.

### G3BP1 ablation does not abolish TAT-RasGAP_317–326_-mediated sensitization of cancer cells to cisplatin

**Figure 8:**
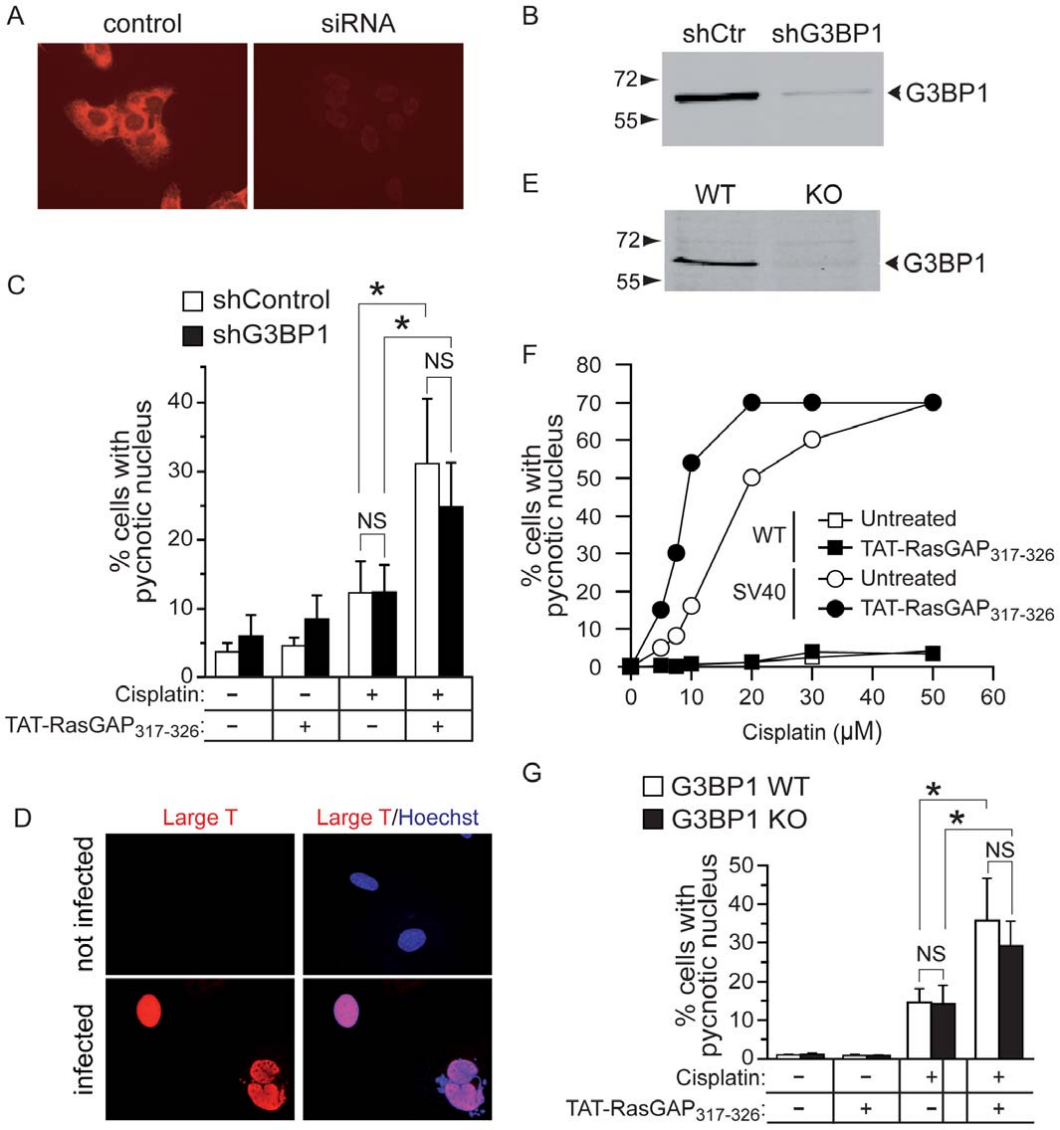
G3BP1 silencing does not affect TAT-RasGAP_317–326_-mediated sensitization of cancer cells to cisplatin-induced apoptosis. **A, B.** U2OS were infected with a non-target shRNA control vector or a G3BP1 shRNA-expressing lentivirus. After 72 hours, cells were analyzed by immunocytochemistry (panel A) and Western blotting (panel B) for the presence of G3BP1. **C.** Infected cells were incubated with 20 µM cisplatin and 20 µM TAT-RasGAP_317–326_ for 22 hours as indicated on the figure. Apoptosis was then scored. **D.** MEFs were infected or not with a large T antigen-expressing lentivirus and 3 days later the expression of the large T antigen was assessed by immunocytochemistry. **E.** G3BP1 expression in wild-type (WT) and G3BP1 knock-out (KO) SV40 large T antigen-transformed MEFs was assessed by Western blotting. **F.** Alternatively, these cells were incubated with increasing concentrations of cisplatin in the presence or in the absence of TAT-RasGAP_317–326_ for 22 hours. Apoptosis was then measured by scoring the number of cells with pycnotic nuclei. **G.** Wild-type (WT) and G3BP1 knock-out (KO) SV40 large T-transformed MEFs were treated as in panel C. Asterisks indicate statistically significant differences; NS: not significant.

The evidence provided so far indicates that G3BP1 is not a (strong) RasGAP or fragment N2 binding partner and that TAT-RasGAP_317–326_ does not modulate any of the known G3BP1 functions. To unequivocally determine whether G3BP1 is needed for TAT-RasGAP_317–326_-mediated tumor sensitization, we used tumor cells in which G3BP1 was silenced and transformed MEFs from G3BP1 knock-out mice. Silencing G3BP1 using shRNA directed at the 3′ UTR of its mRNA resulted in 90% reduction in G3BP1 levels in U2OS cells (Figure 8A–B). This however did not prevent TAT-RasGAP_317–326_ from sensitizing the cells to cisplatin-induced death (Figure 8C). Earlier work has demonstrated that TAT-RasGAP_317–326_ does not sensitize non-cancer cells to cisplatin-induced apoptosis [2]. MEFs, which are non-cancer cells, were indeed not experiencing more cisplatin-induced death in presence of the peptide (Figure 8F). It is however possible to transform MEFs with the SV40 large T antigen [38]. We therefore expressed the large T antigen in MEFs via lentiviral infection (Figure 8D) and, as expected, this rendered them sensitive to the genotoxin-sensitizing effect of TAT-RasGAP_317–326_ (Figure 8F). However, the peptide displayed identical sensitizing efficacy in large T-transformed MEFs lacking or not G3BP1 (Figure 8G). G3BP1 is therefore dispensable for TAT-RasGAP_317–326_ to mediate its genotoxin-sensitizing effect on cancer cells.

## Discussion

The tumor-sensitizing activity of fragment N2 towards genotoxin-induced apoptosis resides in a 10 amino acid stretch corresponding to amino acids 317–326 of RasGAP [2]. This peptidic sequence fused to a cell-permeable peptide (the so-called TAT-RasGAP_317–326_ peptide) is indeed capable of favoring the death of several tumor cell lines to various genotoxins [2]. Amino acids 317–326 of RasGAP correspond exactly to the sequence reported to mediate the binding of RasGAP to G3BP1 [8]. G3BP1 is a protein regulating mRNA stability, stress granule formation, and other cellular functions (reviewed in [39]). Formation of stress granules in cells has been reported to inhibit apoptosis [17]. Additionally, G3BP1 is over-expressed in certain tumors such as breast cancers [40]. As G3BP1 binds to amino acid 317–326 of RasGAP, an attractive hypothesis to explain how TAT-RasGAP_317–326_ sensitizes specifically tumor cells to genotoxin-induced death was that the peptide inhibits the ability of G3BP1 to form stress granule and consequently, as stress granules may exert anti-apoptotic properties [16], decreases the resistance of cancer cells towards apoptosis. This hypothesis would predict that cells lacking G3BP1 would not be sensitized by TAT-RasGAP_317–326_. The evidence reported in the present study demonstrates that this hypothesis is incorrect. First, genotoxins did not induce the formation of stress granules in cancer cells. Formation of stress granules cannot therefore represent a protective mechanism against genotoxins in these cells. Secondly, TAT-RasGAP_317–326_ did not modulate stress granules in conditions known to induce their formation (e.g. in the presence of arsenite). Third, the peptide efficiently sensitizes tumor cells lacking G3BP1 to genotoxin-induced death. It can therefore be unequivocally concluded that G3BP1 plays no role in the anti-tumor activity of TAT-RasGAP_317–326_.

The non-implication of G3BP1 in the function of TAT-RasGAP_317–326_ led us to reassess the reported interactions of G3BP1 with RasGAP. The RasGAP-G3BP1 interaction was first reported in 1996 [8]. In this report, it was shown by Far Western blotting that a fusion protein between GST and the SH3 domain of RasGAP bound to G3BP1 from ER22 cell lysates and that this binding could be prevented by the addition of a peptide corresponding to the 317–326 RasGAP amino acid sequence. We were not able to reproduce these data using U2OS cell lysates and a recombinant histidine-tagged SH3 domain of RasGAP as a probe in the Far Western blotting procedure (data not shown). Moreover, in conditions where RasGAP and G3BP1 bound to known partners (i.e. p190 RhoGAP and USP10, respectively), no interaction between G3BP1 and RasGAP was detected (Figures 1, 2, 3). We used exponentially growing cells for these experiments as it was reported that G3BP1 does not interact with RasGAP in quiescent cells [9]. The results presented in Figures 1, 2, and 3 contrast with reports showing binding of RasGAP to G3BP1 by co-immunoprecipitation methods [8], [9] and by using GST pull-down assays [10]. It has to be noted however that these studies did not provide controls excluding a non-specific binding to beads for example. The interaction between RasGAP and G3BP1 might be occurring in very specific situations. It has been reported that G3BP1 only binds to serum-stimulated cells [8], [9] and only after specific cyclical periods of time following the stimulation: association detected 1 hour, 8 hours and 16 hours after serum addition but no association seen 10 minutes, 20 minutes, 2 hours, 4 hours following serum addition [9]. Whether such pattern of G3BP1-RasGAP association correlates with a known physiological cell cycle has not been defined.

**Figure 9:**
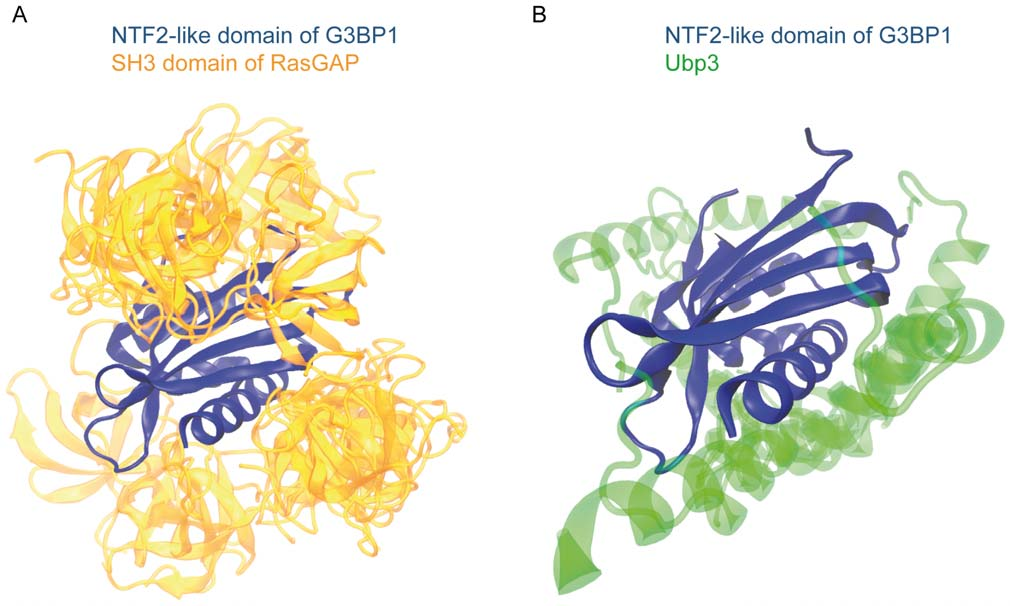
*In silico* assessment of G3BP1 binding to RasGAP. *In silico* docking assays were performed as described in the methods with the SH3 domain of RasGAP on either the G3BP1 NTF2-like domain (panel A) or sequence 190–233 of Ubp5, the yeast orthologue of USP10 (panel B). The 15 best interactions are presented in the figure. While the interactions of G3BP1 and USP10 were of similar nature (note the superposition of the USP10 structures in green), there was no preferred docking interaction between the SH3 domain of RasGAP and G3BP1.

We also tried to crosslink RasGAP to G3BP1 in U2OS cell lysates to visualize a 180 kDa RasGAP-G3BP1 complex, as previously shown [9], but our analysis failed to reveal such a complex (data not shown). We were therefore unable to reveal a binding between G3BP1 and either RasGAP or the SH3 domain-containing fragment N2 of RasGAP, even when we used techniques, conditions (e.g. cells incubated with serum) and cell lines (e.g. CCL39) used by others to report this interaction. A similar lack of interaction between RasGAP and G3BP1 has been reported earlier by another laboratory [12]. It is pertinent to mention here that RasGAP was not found in the proteins identified by mass spectrometry in the cellular material pulled down with an anti-G3BP1 antibody (Sophie Martin and Jamal Tazi, unpublished results). The reverse was also true, i.e. G3BP1 was not identified by mass spectrometry in the cellular material pulled down with an anti-RasGAP antibody (Hadi Khalil and Christian Widmann, unpublished results). Last, we took advantage of the recently crystallized structure of the NTF2 domain of human G3BP1 (PDB; http://www.pdb.org; #3QN) and the previously published structure of the RasGAP SH3 domain [41] to conduct molecular dynamics and docking simulations. This analysis failed to reveal a preferred binding site between the NTF2 domain of G3BP1 and the SH3 domain of RasGAP (Figure 9A). As a control, the ability of USP10 to dock to G3BP1 was also tested. Figure 9B shows that there was a preferred interaction conformation between these two proteins at sites demonstrated to interact in the crystal structure made of these two proteins [42]. In light of all our results, the possibility that RasGAP is not a genuine G3BP1 partner has to be considered.
